# Ethyltoluenes Regulate Inflammatory and Cell Fibrosis Signaling in the Liver Cell Model

**DOI:** 10.3390/toxics12120856

**Published:** 2024-11-27

**Authors:** Suryakant Niture, Sashi Gadi, Hieu Hoang, Leslimar Rios-Colon, Wanda Bodnar, Keith E. Levine, Deepak Kumar

**Affiliations:** 1 The Julius L. Chambers Biomedical/Biotechnology Research Institute (JLC-BBRI), North Carolina Central University (NCCU), Durham, NC 27707, USA; 2 NCCU-RTI Center for Applied Research in Environmental Sciences (CARES), RTI International, Durham, NC 27707, USA

**Keywords:** ethyltoluenes, cell proliferation, inflammation, steatosis, fibrosis, liver cell models

## Abstract

Crude oil naphtha fraction C9 alkylbenzenes consist of trimethylbenzenes, ethyltoluenes, cumene, and n-propylbenzene. The major fraction of C9 alkylbenzenes is ethyltoluenes (ETs) consisting of three isomers: 2-ethyltoluene (2-ET), 3-ethyltoluene (3-ET), and 4-ethyltoluene (4-ET). Occupational and environmental exposure to ETs can occur via inhalation and ingestion and cause several health problems. Exposure to ETs causes eye and upper respiratory tract irritation, coughing, gagging, vomiting, griping, diarrhea, distress, and depressed respiration. Previous studies suggest that ETs target the respiratory tract and liver and produce several lesions in the nose, lungs, and liver areas. In the current study, we investigated the impact of low concentrations of ETs on cell metabolism, cell inflammation, steatosis, and fibrosis signaling in liver cell models in vitro. Dose-dependent exposure of 2-ET, 3-ET, and 4-ET to HepaRG and hepatocellular carcinoma (HCC) HepG2 and SK-Hep1 cells affects cell survival/real-time proliferation and increases ROS production. ETs induce inflammatory *CAT, SOD1, CXCL8, IL1B, HMOX1, NAT1 (3),* and *STAT3* gene expression. Exposure of 2-ET, 3-ET, and 4-ET to HepaRG and HCC HepG2 and SK-Hep1 cells affects mitochondrial respiration/cellular energetics and upregulates metabolic *CYP1-A1, CYP1-A2, CYP2-D6, CYP2-E1, CYP3-A4, CYP3-B4,* and *VEGFA* gene expression. However, no significant change in lipogenesis-related gene expression and modulation of cell steatosis was observed after ET exposure. Acute exposure to induvial ETs and in combination or chronic 2-ET exposure alone modulates cell fibrosis markers such as *AST, FGF-23, Cyt-7 p21, TGFβ, TIMP2,* and *MMP2* in liver cell models, suggesting that ETs target liver cells and may dysregulate liver function.

## 1. Introduction

The crude oil naphtha fraction contains 9-carbon isomers (C9) alkylbenzenes and largely contained ethyltoluenes (ETs) (CH_3_C_6_H_4_CH_2_CH_3_), 1,2,4-trimethylbenzene, 1,3,5-trimethylbenzene, 1,2,3-trimethylbenzene, cumene, and n-propylbenzene. The estimated production of naphtha in the U.S. was 5–10 billion pounds per year from 2012 to 2015 (EPA; CAS 64742-95-6), thus making many C9 alkylbenzenes high production. Despite its high prevalence in the environment, some of these C9 alkylbenzene’s toxicity has not been analyzed. Ethyltoluenes (ETs) are organic aromatic hydrocarbons, and there are three ET isomers exist: 2-ethyltoluene (2-ET), 3-ethyltoluene (3-ET), and 4-ethyltoluene (4-ET) (the ring bears two substituents: a methyl group and an ethyl group) [1]. The ethylation of toluene produces ET. ETs are colorless liquids with similar boiling points. These organic hydrocarbons are used to produce many industrial products such as polystyrenes, rubber, dyes, pesticides, and petrochemicals [2]. These flammable agents, such as 2-ET, 3-ET, and 4-ET vapors, are heavier than air, may travel significant distances, and act as the source of ignition. During exposure, ETs cause dry skin, eye and upper respiratory tract irritation, and respiratory arrest and are involved in rapidly developing pulmonary edema [3]. ETs catalyze oxidation, nitration, and sulfonation reactions and react exothermically with bases and diazo compounds (United States Costal Guard, 1999). The exact half-life of ET isomers is still unknown; however, the half-life for the structurally similar o-ethyltoluene is 4.9 h, 1,3,5-trimethylbenzene is 5.4 h [4], and the ET precursor toluene half-life is 13 h in the atmosphere, as reported by the Agency for Toxic Substances and Disease Registry (ATSDR).

In the general population, ET exposure most likely occurs via inhalation and is typically lower than occupational exposure (ATSDR). Oral exposure may be possible via the consumption of contaminated drinking water, and dermal exposure may be possible through the handling of gasoline or consumer products [5]. Depending on the industry, occupational exposure to C9 alkylbenzenes occurs primarily through inhalation and dermal absorption, with exposures ranging from 0 to 3 ppm (15 mg/m^3^) [6]. In 1985, according to the Toxic Substances Control Act (TSCA), Section 4(a) mandated biological toxicity testing of the C9 fraction of crude oil to analyze the impact of C9 fraction on mutagenicity, neurotoxicity, reproductive toxicity, and inhalation toxicity. Since isomers of ethyltoluene (2-ET, 3-ET, and 4-ET) are the major portion of C9 fraction, in 2009, as part of EPA’s High Production Volume (HPV) Challenge Program, the hazardous exposure effect of 4-ET was investigated, and no evidence of mutagenicity was observed in vitro. However, in the oral gavage study, repetitive administration of 4-ET (300 mg/kg/day) for 13 weeks to male and female rats, changes in body weight, hematological parameters, testicular atrophy, clinical chemistry, and decreased spermatogenesis were reported. Inhalation of 4-ET at a concentration of 979 ppm in male and female rats reduced gonad weight and significantly increased liver weights reported (https://ntp.niehs.nih.gov/sites/default/files/ntp/about_ntp/bsc/2014/june/alkylbenzenes_concept_508.pdf; accessed on 23 March 2024), indicating that 4-ET may dysregulate liver function in rats. On the other hand, oral gavage exposure to 4-ET shows developmental toxicity, changes in liver weight, leukocyte differentials, and clinical pathology (International Research and Development Corporation 1980-81). Inhalation exposure to 4-ET was found to cause mild skin and eye irritancy and changes in the number of macrophages, leukocytes, and lymphocytes found in bronchoalveolar lavage [7]. Recently, comparative inhalation toxicity of various ET isomers was reported, and the study revealed that 2-ET causes maximum damage in the nose, lungs, and liver in the animals compared with 3-ET and 4-ET [8]. Importantly, nasal inhalation of 2-ET induced hepatocellular hypertrophy and necrosis in animals [8], whereas a whole-body inhalation exposure to 2-ET produced adverse clinical symptoms. 2-ET exposure increased acute toxicity in rats and mice, decreased survival and body weight, and diffuse nasal olfactory epithelial degeneration in rats or necrosis in mice [6]. Exposure to 150 and 300 ppm of 2-ET, atrophy of the olfactory epithelium and nerves was observed in all animals (rats and mice), whereas these lesions were more severe in mice than in rats [6].

Since nasal inhalation or whole-body inhalation exposure to ETs causes liver necrosis [8], in the current study, we aimed to determine the relative impact of ETs on liver cytotoxicity. In this report, we analyzed the direct effect of low concentrations of ETs on cell survival/real-time proliferation, metabolism, inflammation, cell steatosis, and fibrosis signaling in liver cell models. Our data suggest that exposure to ETs upregulated ROS production, induced inflammatory and cell metabolic gene expression, and modulated cell fibrosis signaling in liver cells.

## 2. The Materials and Methods

### 2.1. Cell Culture

Human immortalized and terminally differentiated HepaRG cells (Cat # HPRGC10) were obtained from ThermoFisher Scientific (Waltham, MA, USA). HepaRG cells were cultured using a specific thawing medium/plating medium (Cat # HPRG770) and expanded in William’s E Medium (Cat #12551032) supplemented with maintenance and metabolism medium (1X) (Cat # HPRG720) and 1% GlutaMax (Cat # 35050061) as per the manufacturer’s instructions. HepaRG cells are fully functional human hepatic cells and exhibit many characteristics of primary human hepatocytes [9]. Hepatocellular carcinoma (HCC) HepG2 (Cat # HB-8065) and SK-Hep1 (Cat # HBT-52) cell lines were obtained from the ATCC. The HepG2 cell line exhibits epithelial-like morphology, and the SK-Hep1 cell line is endothelial. HepG2 and SK-Hep1 cells were grown in Eagle’s minimum essential medium (EMEM) (ATCC; Cat # 30-2003) supplemented with 10% Fetal Bovine Serum (FBS; Access Biologicals, Vista, CA, USA) and 50 U/mL penicillin/streptomycin (Cellgro, Technologies, Lincoln, NE, USA). Cells were incubated at 37 °C in a cell culture incubator with 5% CO_2_.

### 2.2. Ethyltoluenes Exposure

Ethyltoluenes (ETs) were obtained from *Tokyo Chemical Industry* Co., Ltd. (Tokyo, Japan) 2-ethyltoluene (2-ET, >99.0% pure, Cat # E0184), 3-ethyltoluene (3-ET, >98% pure, Cat # E0185), and 4-ethyltoluene (4-ET, >97% pure, Cat #E0186) were obtained. We prepared serial dilutions of ETs in respective cell culture medium, and after vigorous vortexing, freshly prepared ETs (50 to 250 nM), as indicated in different experiments, were exposed to HepaRG and HCC cells. We generated the 2-ET-tolerant HepaRG and HepG2 cells by exposing the cells to 50 nM of 2-ET twice weekly and by trypsinization and passaging the cells for 40 days in the presence of 2-ET. Untreated control HepaRG and HepG2 cells were similarly sub-cultured without 2-ET exposure.

### 2.3. MTT Cell Survival Assay

HepaRG and HCC HepG2 and SK-Hep1 cells were plated in 96-well plates (Falcon, Corning, NY, USA) (5000 cells/well) and incubated overnight, and cells were exposed to 1 nM to 1 mM concentrations of ETs for 72  h. A colorimetric cell survival MTT (3-(4,5-dimethylthiazol-2-yl)-2,5-diphenyl tetrazolium bromide) assay was performed. MTT reagent was obtained from MP Biomedicals (Solon, OH, USA). After 72 h of exposure to ETs, cells were treated with MTT reagent in medium (5 μL/well; stock—5 mg/mL in PBS) and incubated for 1 h at 37 °C. After PBS wash, formazan crystals produced by cells were dissolved in DMSO (Fisher chemical, 99.9%), and color intensity was measured by reading the plate at 570  nm using a FLUOstar^®^ Omega microplate reader (BMG Lab Tech, Cary, NC, USA). All experiments were repeated three times in triplicates.

### 2.4. Real-Time Cell Proliferation

HepaRG and HCC HepG2 and SK-Hep1 cells were plated in 96-well plates (Falcon, Corning, NY, USA) (2500 cells/well) for 12 h, and then cells were exposed to 1 nM to 1 mM of ET concentrations for 0 to 120 h in an incubator supplied with 5% CO_2_. Incucyte (Sartorius) live-cell imaging system was used to analyze real-time cell proliferation.

### 2.5. ROS Quantification

HepaRG and HepG2 cells (5000 cells/well) were plated in 96-well plates (Costar- 39120) in triplicate overnight, and cells were treated with 50–250 nM of ETs. After 72  h, cells were treated with CellROX Green Reagent (Thermo Fisher; Cat # C10444) as per manufacture instruction in a complete medium at 37 °C for 30 min. The cells were then washed with PBS and observed under a Keyence BZX-810 fluorescence microscope (10× objective), and images were captured. Similarly, the cellular endogenous ROS-related green fluorescence was quantified after treatments with ETs by using the FLUOstar^®^ Omega plate reader using excitation/emission at 485/520 nm and plotted.

### 2.6. Seahorse Bio-Analyzer

The Cell Mito-Stress assay was used to determine the effect of ETs on mitochondrial oxidative phosphorylation (OXPHOS) and cell energy phenotype in HepaRG, HepG2, and SK-Hep1 cells. Cells were seeded at a density of 2 × 10^4^ cells/well in XFp plates, allowed to grow overnight, and treated with 50 nM of ETs for 72 h. After 72 h, cells were incubated with XF media supplemented with 10 mM D-glucose and 2 mM L-glutamine, as described in the manufacturer’s instructions. Extracellular acidification rate (ECAR) and oxygen consumption rate (OCR) were measured using a Seahorse XFp analyzer. For HepaRG, HepG2, and SK-Hep1 cells, injections of 1 μM Oligomycin, 0.5 μM FCCP [Carbonyl cyanide-4 (trifluoromethoxy) phenylhydrazone], and 0.5 μM Rotenone were simultaneously applied to measure glycolysis and OXPHOS in the cells. The results were analyzed using Wave software version 2.6 (Seahorse/Agilent, Santa Clara, CA, USA).

### 2.7. Immunoblotting

Immunoblotting was performed as described previously [10]. In brief, HepaRG and HepG2 (1  ×  10^5^) were grown in 6-well plates overnight. Cells were exposed to indicated concentrations of ETs for 72  h. Cells were washed with cold PBS and lysed in cell lysis buffer (Cell Signaling, Danvers, MA, USA) containing protease inhibitor mixture (Roche, Indianapolis, IN, USA) and 0.1 mM PMSF (phenylmethylsulfonyl fluoride). After centrifugation at 10,000  rpm for 10  min, supernatants were used for protein quantification. Bio-Rad protein assay dye (Bio-Rad, Hercules, CA, USA, Cat # 5000006) was used. For immunoblotting, equal amounts of proteins (60 μg) were separated on NuPAGE 4%–12% Bis-Tris-SDS gel (Invitrogen, Carlsbad, CA, USA). The proteins were transferred to a polyvinylidene difluoride (PVDF) membrane (Thermo Scientific, Rockford, IL, USA). After blotting, the PVDF membranes were blocked in 1× blocking buffer (Sigma-Aldrich, St. Louis, MO) for 1 h and incubated with indicated primary antibodies (1:1000 dilution) overnight at 4 °C. The primary antibodies were purchased from Cell Signaling Technology (Danvers, MA, USA): anti-p21 (Cat #2947s), anti-TIMP2 (Cat #5738s), anti-MMP2 (Cat #13132s), anti-TGFβ (Cat #3711s), and anti-β-tubulin (Cat # 2128S). After incubation of primary antibodies, the membranes were washed with Tris-buffered saline with 0.1% Tween 20 (TBST, Sigma-Aldrich, St. Louis, MO, USA). The membranes were further incubated in the appropriate secondary antibody (1:10000 dilution) (Jackson ImmunoResearch, West Grove, PA, USA) for 1 h at room temperature. The immunoblots were developed using ECL chemiluminescence detection reagents (Signagen Laboratories, Rockville, MD, USA). The Western blots were developed using the Azure C-500 Bio-system.

### 2.8. RT/qPCR

HepaRG and HepG2 cells (1  ×  10^5^) were plated in 6-well plates overnight. Cells were treated with the indicated concentrations of ETs for 72 h. Cells were washed with cold PBS, and total RNA from control and ET-treated cells were isolated using the TRIZOL reagent (Invitrogen, Carlsbad, CA, USA). One microgram of RNA was reverse transcribed using a High-Capacity cDNA Reverse Transcription kit (Applied Biosystems, Carlsbad, CA, USA), and cDNA was mixed with Power SYBR Green PCR master mix (Applied Biosystems) with both specific forward and reverse human primers of the inflammatory signaling genes, cellular drug/xenobiotic metabolic genes, lipogenesis-related genes and cell fibrosis signaling genes (Supplementary Table S1). All primers were obtained from Integrated DNA Technology (IDT, Coralville, IA, USA). A human *GAPDH* forward and reverse primers used to express the *GAPDH* gene as an internal control. The PCR mixtures were run on a QuantStudio-3 PCR System (Applied Biosystems) using relative quantitation according to the manufacturer’s protocols.

### 2.9. Oil Red O Staining and Oil Red O Staining-Based Cell Steatosis Quantification

HepaRG, HepG2, and SK-Hep1 cells (1  ×  10^4^) were grown on coverslips in six-well plates and treated with 2-ET, 3-ET, and 4-ET (50 nM). After 48 h, cells were exposed to 100  μM oleic acid (Sigma) for induction of cellular steatosis for 24 h in the presence of ETs. Cells were washed with PBS, fixed with paraformaldehyde (4%), and stained with Oil Red O (ORO) staining as described previously [11]. ORO-stained cell images were captured using a Nikon Y-IDP microscope. In another experiment, Oil Red O staining-based steatosis quantification was performed [12]. HepaRG, HepG2, and SK-Hep1 cells (1  ×  10^4^/well) were grown in 6-well plates in triplicate and treated with ETs for 48 h and oleic acid for 24 h as indicated. After ORO staining, cells were lysed, lysates (100 µL) were transferred to a 96-well plate, and the plates were read at 405 nm. All experiments were repeated two times in triplicates.

### 2.10. Statistical Analysis

Results are from independent experiments (n = 3) and presented as means  ±  SEM. Differences between groups were analyzed using either a two-tailed Student’s *t*-test or one-way ANOVA followed by Tukey HSD post hoc test. A *p*-value of <0.05 was considered statistically significant. Statistical significance between means was determined by Graph Pad Prism 9 software version 10.4.0 (GraphPad Software Inc., La Jolla, CA, USA).

## 3. Results

### 3.1. ETs Affect Cell Survival and Proliferation in Liver Cell Models

**Figure 1:**
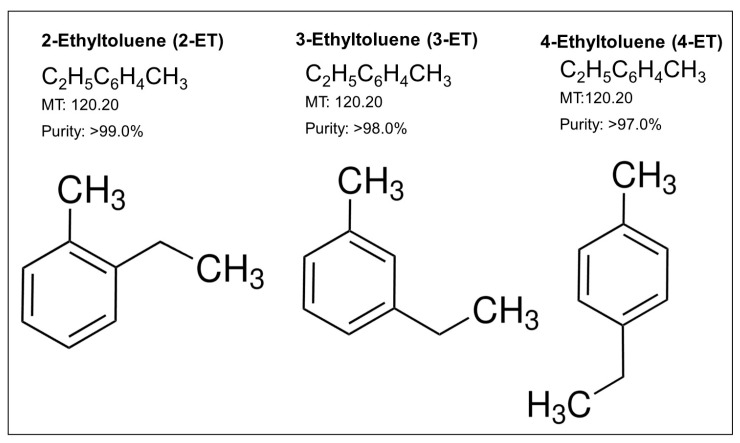
Chemical structures of ETs. The molecular weights and purities of ETs used in this study are presented.

Carbon 9-alkylbenzenes consist of ethyltoluene isomers such as 2-ethyltoluene (2-ET), 3-ethyltoluene (3-ET), and 4-ethyltoluene (4-ET) aromatic organic compounds. We obtained these isomers with high purity (>98%). These isomers show the same molecular weights. However, methyl groups are associated with different carbons: 2-ET: 1-ethyl-2-methylbenzene, 3-ET: 1-ethyl-3-methylbenzene, and 4-ET: 1-ethyl-4-methylbenzene (Figure 1).

**Figure 2:**
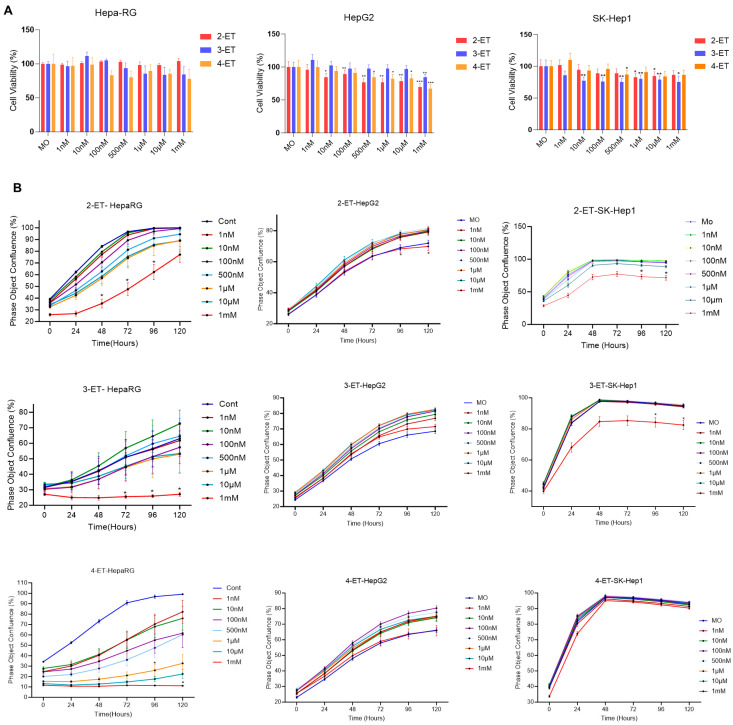
**ETs affect cell proliferation in liver cells.** (**A**) HepaRG and HCC HepG2 and SK-Hep1 cells were cultured in 96-well plates (5000 cells/well), and after 16 h, cells were exposed to 1 nM to 1 mM concentrations of ETs for 72 h. MTT cell viability assay was performed, and relative cell survival was analyzed (left, middle, and right panels). * *p* < 0.05, ** *p* < 0.01, *** *p* < 0.001 compared to the control cells. (**B**) HepaRG, HepG2, and SK-Hep1 cells cultured in 96-well plates (2500 cells/well) and treated with 1 nM to 1 mM concentrations of 2-ET, 3-ET, and 4-ET, and the effect of ETs on real-time cell proliferation was analyzed by Incucyte (Sartorius). * *p* < 0.05 compared with control (MO) cells.

ETs are emerging environmental pollutants; earlier studies indicated that direct inhalation of 2-ET in rats and mice produces lesions in the nose and liver and liver necrosis in mice [6,8]. In the current study, we analyzed the impact of low concentrations of ETs on human liver cell survival and proliferation. For this, we utilized terminally differentiated human bipotent progenitor HepaRG liver cells [9] (these cells maintain many characteristics of primary human hepatocytes) and human hepatocellular carcinoma (HCC) cell lines HepG2 and SK-Hep1. Cell survival MTT assay demonstrated that a dose-dependent (1 nM to 1 mM) exposure to 2-ET, 3-ET, and 4-ET decreased average cell survival in HepaRG cells (not significantly) (Figure 2A, left panel), whereas 2-ET, 3-ET, and 4-ET significantly decreased cell survival (20 to 35%) in HepG2 cells exposed with higher concentrations (100 nM to 1 mM) of ETs. Interestingly, 2-ET at 10 nM concentration significantly decreased cell survival in HepG2 HCC cells (Figure 2A, middle panel). In HCC, SK-Hep1 cells, 2-ET, and 3-ET also significantly decreased cell survival, suggesting that ETs affect cell survival in liver cells (Figure 2A, right panel). Further, we analyzed the impact of ETs on the real-time proliferation in HepaRG and HCC cells using an Incucyte (Sartorius) live-cell imager. Exposure of HepaRG to 2-ET, 3-ET, and 4-ET decreased cell proliferation at high concentrations (100 nM to 1 mM) (Figure 2B, left panels). In contrast, exposure of HepG2 cells to 2-ET, 3-ET, and 4-ET (1 nM to 10 µM) showed increased cell proliferation compared to untreated cells (Figure 2B, middle panels). Exposure of 2-ET to SK-Hep1 cells significantly reduced cell proliferation; however, no significant effect on cell proliferation was observed when cells were exposed to 3-ET and 4-ET at 1 nM to 10 µM concentrations (Figure 2B, right panels). Our data suggest that ETs differentially affect cell survival and cell proliferative activities in liver cell models.

### 3.2. ETs Increase Inflammatory Signaling in Liver Cell Model

**Figure 3:**
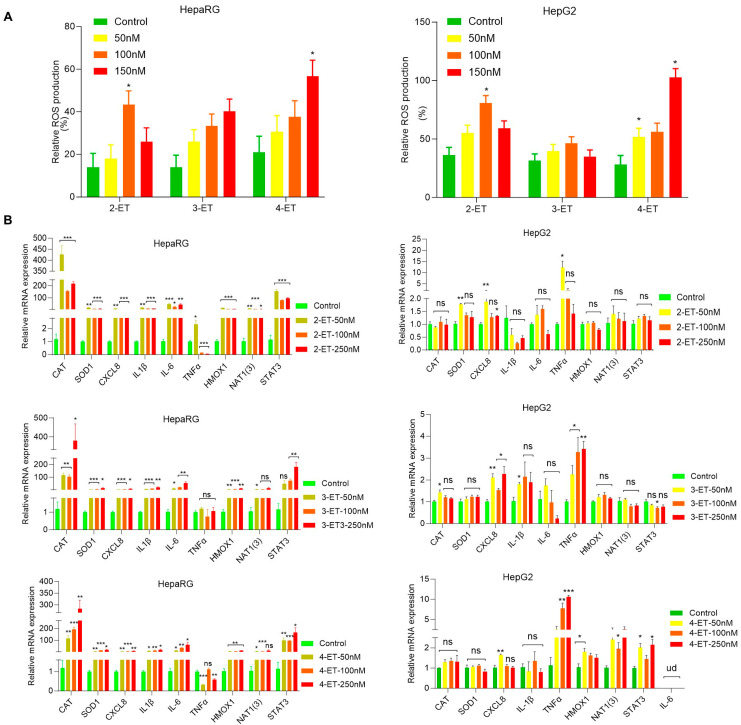
**ET exposure induced reactive oxygen production and inflammatory signaling in liver cells.** (**A**) HepaRG and HepG2 cells were cultured in 96-well plates in triplicates for 12 h, and cells were further treated with ETs (50-250 nM) as indicated for 72  h. The endogenous ROS-related green fluorescence was quantified using the FLUOstar^®^ Omega plate reader using excitation/emission at 485  nm/520  nm and plotted. * *p*  <  0.05, compared with control cells. (**B**) RT/qPCR: HepaRG and HepG2 cells were treated with the indicated concentration of ETs for 72  h. The expressions of inflammatory genes were analyzed by RT/qPCR as described in the Materials and Methods section. * *p*  <  0.05, ** *p*  <  0.01, and *** *p*  <  0.001 compared with untreated cells. ns—not significant.

Since earlier studies indicated that ETs induced neutrophilic inflammation and liver necrosis [8], here, we analyzed the effect of 2-ET, 3-ET, and 4-ET on the production of endogenous reactive oxygen species (ROS). HepaRG and HepG2 cells were exposed to 2-ET, 3-ET, and 4-ET (50 to 150 nM), and cellular ROS production was analyzed after exposure of cells with CellROX Green Reagent (Supplement Figure S1). Cell immunofluorescence intensity data suggest that 2-ET and 4-ET induced ROS production significantly in HepaRG and HepG2 cells (Figure 3A, left and right panels). Further, we analyzed inflammatory gene signatures by RT/qPCR in HepaRG cells after exposure to ETs. Compared with vehicle exposure, 2-ET, 3-ET, and 4-ET induced expressions of *CAT, SOD1, CXCL8, IL1B, IL-6 HMOX1, NAT1(3),* and *STAT3* several folds (Figure 3B, left panels). ETs such as 3-ET and 4-ET reduced pro-inflammatory cytokines *TNF-α* expression in HepaRG cells. In HCC HepG2 cells, 2-ET increased TNF-α, SOD1, and CXCL8 expression, and the expression of TNF-α was also induced after exposure to 3-ET and 4-ET (Figure 3B, right panels), suggesting that ETs upregulate inflammatory signaling in HepaRG cells more aggressively compared with HCC HepG2 cell model (Figure 3B, left and right panels). Collectively, our data suggest that ETs increased cellular ROS production and upregulated cellular inflammatory gene signature in liver cells.

### 3.3. ETs Modulate Mitochondrial Respiration in Liver Cell Model

Since ETs increase cellular inflammation and modulate cell metabolic activities, we further analyzed ETs’ impact on mitochondrial oxygen consumption rate (OCR) in live liver cells by Seahorse in HepaRG and HCC HepG2 and SK-Hep1 cells (Supplementary Figure S2A–C). HepaRG and HCC HepG2 and SK-Hep1 cells were exposed with 2-ET, 3-ET, and 4-ET (50 nM) for 72 h, and mitochondrial oxygen consumption rate (OCR) and extracellular acidification rate (ECAR) were measured by Seahorse. Exposure to 2-ET decreases average OCR and ECAR by 15 to 30% in HepaRG and HepG2 cells (no significant), and no significant effect on OCR and ECAR was observed in SK-Hep1 cells (Supplementary Figure S2A–C, upper panels). Exposure to 3-ET does not show any change in OCR and ECAR in HepaRG, HepG2, or SK-Hep1 cells (Supplementary Figure S2A–C, middle panels). Exposure to 4-ET decreased OCR and ECAR in HepaRG, HepG2, and SK-Hep1 cells (Supplementary Figure S2A–C, lower panels). These data suggest that low concentration of ETs modulates mitochondrial respiration/energetics in a liver cell model.

### 3.4. ETs Increase Cell Metabolic Signaling in Liver Cell Model

**Figure 4:**
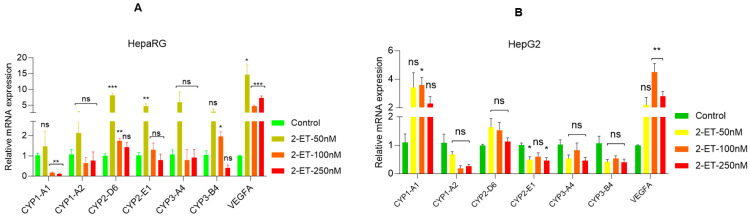

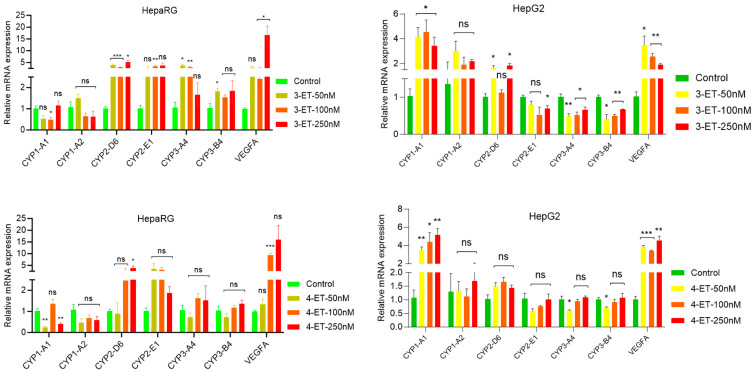
ET exposure modulates drug metabolic gene expression in liver cells. (**A**,**B**) HepaRG and HepG2 cells were treated with the indicated concentrations of ETs (50 nM–250 nM) for 72  h. The drug metabolic gene expressions were analyzed by RT/qPCR as described in the Materials and Methods section. * *p*  <  0.05, ** *p*  <  0.01, and *** *p*  <  0.001 compared with untreated cells. ns—not significant.

Our data suggest that even at low concentrations, ETs regulate cell survival and inflammatory signaling; we ask what role ETs play in cellular metabolism. Since drug/xenobiotic metabolism occurs in the liver and cytochrome P450 (CYP) family enzymes play a key role in the metabolism of drugs and other xenobiotics [13], here, we analyzed the effect of ETs on the regulation of metabolic (*CYP1-A1, CYP1-A2 CYP2-D6, CYP2-E1, CYP3-A4, CYP3-B4,* and *VEGFA*) gene expression in liver cells. Exposure to 2-ET, even at 10 nM concentration, significantly increased *CYP1-A2, CYP2-D6, CYP2-E1, CYP3-A4, CYP3-B4,* and *VEGFA* gene expression in HepaRG cells and *CYP1-A1* and *VEGFA* in HepG2 cells (Figure 4A,B, upper panels). Exposure to 3-ET significantly increased *CYP2-D6, CYP2-E1, CYP3-A4, CYP3-B4,* and *VEGFA* gene HepaRG cells and *CYP1-A1, CYP2-D6,* and *VEGFA* genes in HepG2 cells (Figure 4A,B, middle panels). Moreover, Exposure to 4-ET significantly increased the *VEGFA* gene in HepaRG cells and *CYP1-A1* and *VEGFA* gene in HepG2 cells (Figure 4A,B, lower panels), suggesting that ETs modulate drug metabolic gene expression in liver cells.

### 3.5. ETs Did Not Affect Lipogenic Signaling in a Liver Cell Model

In the liver, nonalcoholic fatty liver disease (NAFLD) and alcoholic fatty liver disease (AFLD) start with the increasing accumulation of lipid droplets (steatosis) in hepatocytes that modulate hepatic inflammation (nonalcoholic steatohepatitis: NASH), which eventually leads to fibrosis, cirrhosis, and HCC [14,15]. There are two-hit models that reveal the important early metabolic events that can cause hepatocellular necrosis in nonalcoholic steatohepatitis (NASH), and 2-ET can cause hepatocellular necrosis, as reported earlier [8]. In the current study, we analyzed the effect of ETs on cell steatosis/lipogenesis. Since hepatic lipogenesis induction is modulated by several lipid/fatty acid metabolic enzymes/proteins [16], we examined the effect of ETs (50 nM) on the expression of lipid/fatty acid metabolic gene expression including fatty acid synthase (*FASN*), liver acetyl-CoA carboxylase, (*ACC*), fatty acid-binding protein-1 (*FABP1*), stearoyl-CoA desaturase-1 (*SCD1*), and transcription factors such as sterol regulatory element-binding protein 1 (*SREBP1*) (Supplement Figure S3A,B). RT/qPCR data suggest that 2-ET treatment significantly increased expression of *SREBP1*, *ACC*, *FABP*, and 4-ET increased expression of *SREBP1* in HepaRG cells (Supplement Figure S3A). In HepG2 cells, 2-ET and 3-ET show increased (not significantly) *SREBP1*, *FASN*, *ACC*, *FABP*, and *SCD1* genes (Supplement Figure S3B). Further, we also analyzed the effect of ETs on oleic acid-mediated cell steatosis in HepaRG cells and HCC HepG2 and SK-Hep1 cells (Supplement Figure S3C). Oil Red O staining demonstrated that ET exposure does not induce cell steatosis in HepaRG or HCC HepG2 and SK-Hep1 (Supplement Figure S3C,D), suggesting that ET exposure did not affect cell steatosis in liver cells.

### 3.6. ETs Increase Cell Fibrosis Signaling in a Liver Cell Model

**Figure 5:**
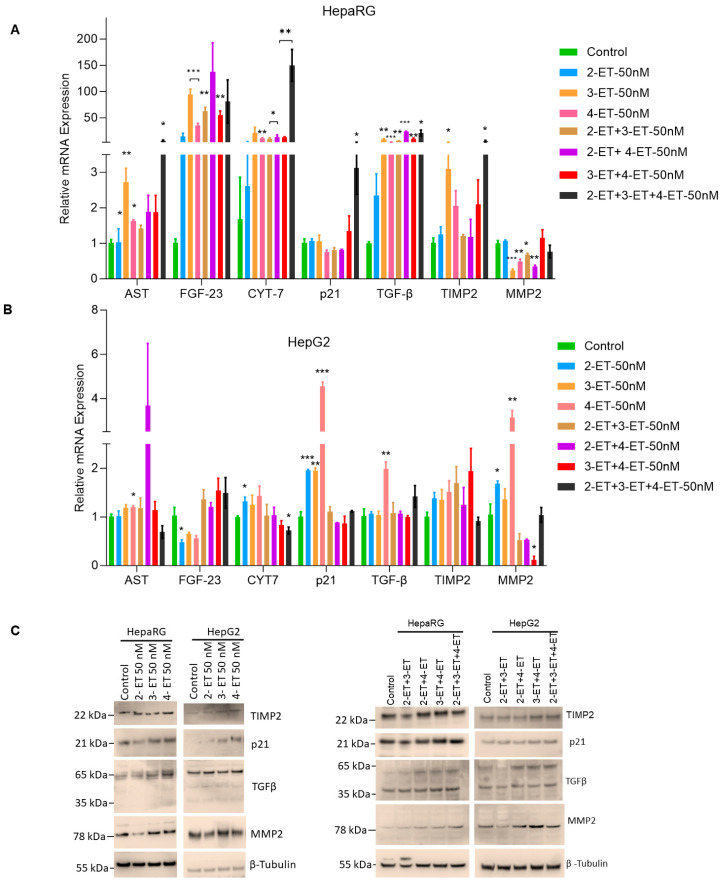
ET exposure increases cell fibrosis in liver cells. (**A**,**B**) HepaRG and HepG2 cells were treated with ETs (50 nM) as indicated for 72  h. The expressions of cell fibrosis genes were analyzed by RT/qPCR as described in the Materials and Methods section. * *p*  <  0.05, ** *p*  <  0.01, and *** *p*  <  0.001 compared with untreated cells. (**C**) The effect of 2-ET, 3-ET, and 4-ET alone or in combination (50 nM each as indicated) on the expression of cell fibrosis proteins was analyzed by immunoblotting.

Although ETs do not regulate cell steatosis but cause inflammation in HepaRG and HCC cells, here, we further investigated the effect of ETs (single exposure and in combination) on the expression of cell fibrosis markers such as *AST*, *FGF-23*, *Cyt-7*, *p21*, *TGF-β*, *TIMP2*, and *MMP2*. RT/qPCR data demonstrated that exposure to 2-ET or in a combination of 3-ET and 4-ET increased *FGF-23*, *Cyt-7*, and *TGF-β* in HepaRG cells (Figure 5A). 3-ET alone or in combination with 2-ET and 4-ET treatments increased *AST, FGF-23*, *Cyt-7*, *TGF-β*, and *TIMP2* gene expression, whereas 4-ET alone or in combination with 2-ET and 3-ET exposure increased expression of *AST, FGF-23*, *Cyt-7*, *p21*, *TGF-β*, and *TIMP2* genes in HepaRG cells (Figure 5A) suggestion that ETs increase cell fibrosis gene expression significantly in HepaRG cells. On the other hand, RT/qPCR data also demonstrated that exposure to 2-ET, 3-ET, and 4-ET alone increased the expression of *Cyt-7*, *p21, TIMP2,* and *MMP2* in HepG2 cells (Figure 5B). 2-ET-alone treatments increased the expression of Cyt1*, p21*, and *MMP2* genes, whereas 4-ET-alone exposure increased the expression of *p21*, *TGF-β,* and *MMP2* in HepG2 cells (Figure 5B). Interestingly, combined exposure ETs, as indicated, do not show increased cell fibrosis gene expression in HepG2 cells. Our data suggest that ETs alone or in combination induced several-fold cell fibrosis-associated gene expressions in HepaRG cells compared with HCC HepG2 cells.

In addition, we analyzed the expression of the TGF-β, MMP2, TIMP2, and p21 cell fibrosis signaling protein markers by Western blotting after exposing the HepaRG and HCC HepG2 cells to 2-ET, 3-ET, and 4-ET alone (Figure 5C, left panels) or in a combination of 2-ET+3-ET, 2-ET+4-ET, 3-ET+4-ET, and 2-ET+3-ET +4-ET as indicated (Figure 5C, right panels). Western blotting data indicated that compared with controls, 3-ET and 4-ET increased the expression of TIMP2, TGFβ, MMP2, and p21 in HepaRG and HepG2 cells (Figure 5C, right panels). Additionally, combined ET exposure also induced TIMP2, TGFβ, MMP2, and p21 expression in HepaRG and HepG2 cells (Figure 5C, left panels). Collectively, our data suggest that ETs activate cell fibrosis signaling in HepaRG and HCC HepG2 cells.

### 3.7. Chronic 2-ET Exposure Increases Cell Fibrosis in Liver Cell Model

**Figure 6:**
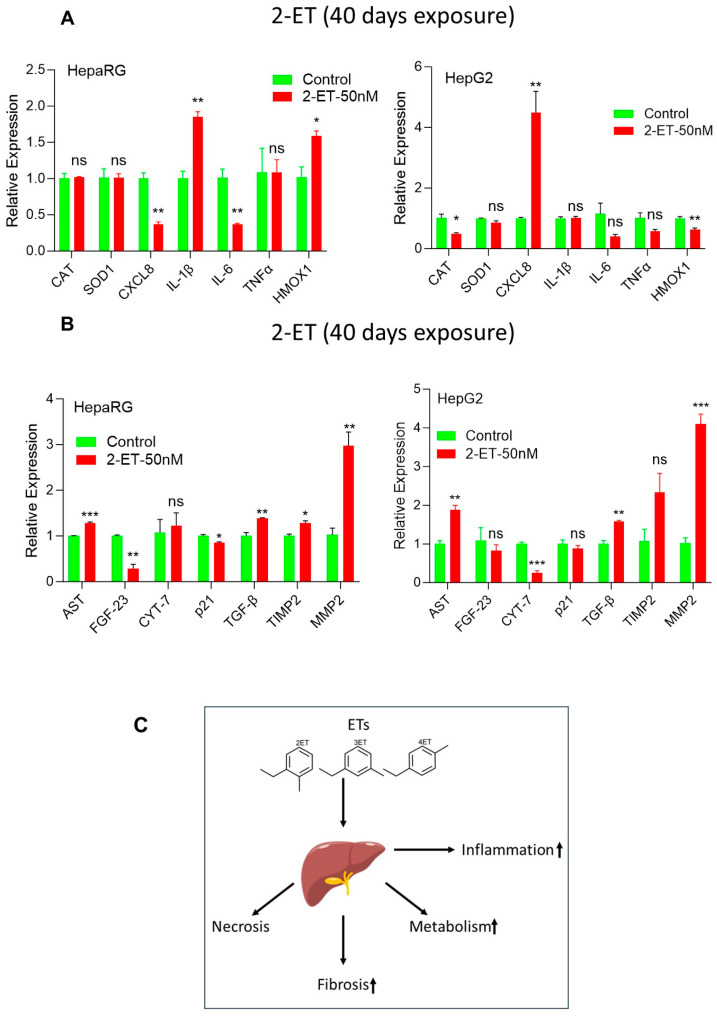
Chronic 2-ET exposure increases liver cell inflammation and cell fibrosis signaling. (**A**,**B**) HepaRG and HepG2 cells were treated with 2-ET (50 nM) for 40 days. The expression of inflammatory genes (**A**) and cell fibrosis genes (**B**) were analyzed by RT/qPCR. * *p*  <  0.05, ** *p*  <  0.01, and *** *p*  <  0.001 compared with untreated cells. (**C**) A schematic representation shows the possible role of ETs in the regulation of inflammation, metabolism, and cell fibrosis signaling in the liver. ns—not significant.

Since 2-ET modulated inflammatory, metabolic, and fibrosis signaling in liver cells, to investigate the biological significance of 2-ET in liver cell inflammation and cell fibrosis, we exposed HepaRG and HCC HepG2 cells to 2-ET at 50 nM concentrations for 40 days, and the expression of inflammatory and cell fibrosis biomarkers was analyzed by RT/qPCR and immunoblotting (Figure 6A,B). Chronic exposure to 2-ET did not show a significant effect on *CAT*, *SOD1*, and *TNFα*, decreased expression of *CXCL8* and *IL-6*, and increased expression of *IL-1B* and *HMOX1* gene expression in HepaRG cells (Figure 6A, left panel). In HepG2 cells, 2-ET-induced expression of the *CXCL8* gene and decreased expression of *CAT* and *HMOX1* (Figure 6A, right panel).

We further analyzed the effect of chronic 2-ET exposure on the expression of cell fibrosis biomarkers. RT/qPCR data suggest that exposure to 2-ET at 50 nM concentrations induced the expression of *AST*, *TGFβ*, *MMP2*, and *TIMP2* in HepaRG cells and *AST*, *TGFβ*, and *MMP2* in HepG2 cells significantly (Figure 6B, left and right panels). Indeed, our data suggest that chronic exposure to 2-ET not only increased inflammatory and cell metabolic signaling but also regulated cell fibrosis signaling in HepaRG and HCC HepG2 cells.

## 4. Discussion

The crude oil naphtha fraction contains several fractions of C8–C10 alkylbenzenes [benzene, toluene, ethylbenzene, xylene (BTEX)]. According to the EPA, BTEX is a major environmental pollutant because of underground leakage from gasoline storage tanks into groundwater. Although most of the BTEX chemical fractions are insoluble in water, they are flammable, toxic, carcinogenic, and affect the immune system, respiratory system, metabolism, and reproductive functioning [17,18]. Several conventional methods were used to eliminate BTEX contamination, for example, absorption, aeration, chemical, and biological oxidation [17,18]. 

Since ETs can be produced by ethylation of toluene, the toxicological profile for toluene was reported earlier by ATSDR (https://www.atsdr.cdc.gov/toxprofiles/tp56.pdf; accessed: 12 July 2024). Exposure of C9 fraction (that includes ET isomers) to bacteria or Chinese Hamster Ovary (CHO) cells in vitro and rat bone marrow cells in vivo was reported. The study indicated that there was no mutagenesis or sister chromatid exchange observed in CHO cells when cells were exposed to the C9 fraction [19]. On the other hand, at 1514 ppm concentration of inhalation exposure of C9 fraction, maternal toxicity was observed in pregnant mice. A decrease in the number of litters with viable fetuses and a decreased number of live fetuses/litter were also observed [20]. Inhalation exposure to C9 fraction at 495 and 1480 ppm decreased fetal body weight in the F3 generation, and no neurotoxicity in male rats was observed at 1320 ppm of C9 fraction exposure for 13 weeks [21]. However, gross neurobehavioral toxicity was observed in pregnant mice following C9 inhalation exposure at 1514 ppm during gestation (10 days) [20]. In addition, chronic inhalation of C9 fraction (373 ppm) for 12 months increased liver and kidney weights in male rats [22]. The study further revealed that macrophage infiltration and alveolar wall thickening with severity as observed in the lung and leiomyoma, lymphoma, and glioblastoma tumors were observed in female and male rats. Although it is not clear, it is due to chronic inhalation of C9 fraction [22]. These studies suggest that ETs containing C9 fraction modulate cytotoxicity in vitro and in vivo.

Toluene inhalation increased serum levels of liver enzymes in rats exposed to 2000 ppm for 48 h, rats exposed to 3000 ppm, 1 h/day for 30 days, and rats exposed to 300 ppm, 6 h/day for 4 weeks [23], suggesting that the precursor of ETs, toluene, can cause liver toxicity. Moreover, in rats, inhalation of toluene (acute, intermediate, or chronic) above 300 ppm for 6–8 h daily increases liver weights and induces hepatic cytochrome P450 levels, which can alter live metabolism and cause liver damage [23]. ETs can cause nasal and liver lesions in animals after inhalation exposure [6,8]. Similarly, an earlier study suggests that inhalation of toluene can cause liver damage and workers who were occupationally exposed to toluene (30 and 350 ppm average) and toluene exposure increased liver serum levels of alkaline phosphatase (AP) [24].

Although 2-ET shows low solubility [74.6 mg/L (25 °C)] and 3-ET and 4-ET are not miscible in water, studies suggest that 2-ET can cause severe damage to nasal, liver, and lungs in animals [6,8], and few reposts suggest the effect of toluene compounds (a precursor of ETs isomer) on liver cell cytotoxicity [25,26], the impact of ET isomers on human liver cell cytotoxicity not studied so far. In the current study, we analyzed the effect of acute exposure to 2-ET, 3-ET, and 4-ET at low concentrations [50 to 250 nM (6 to 30 ppb)] on liver cells. Direct exposure to 2-ET, 3-ET, and 4-ET affects cell proliferation and induces inflammatory singling, as well as cell fibrosis in liver cells. A dose-dependent direct exposure of 2-ET, 3-ET, and 4-ET to liver HepaRG cells, HCC HepG2, and SK-Hep1 cells decreased cell survival/real-time proliferation, whereas 50 nm to 250 nM concentration increased ROS production. ETs also induce inflammatory gene expression in liver cells, affecting mitochondrial respiration and cellular energetics. ET exposure affects cell fibrosis gene expression in HepaRG and HCC HepG2 cells. Acute exposure of ETs and 2-ET chronic exposure upregulated cell fibrosis markers such as *AST, FGF-23, Cyt-7 p21, TGFβ, TIMP2,* and *MMP2* in liver cell models indicated that ETs target liver cells and increase cytotoxicity.

The liver may be a relevant target organ as C9 alkylbenzenes are known to be metabolized by P450 enzymes in the liver and our data suggest that exposure to ETs upregulates *CYP1-A2, CYP2-D6, CYP2-E1, CYP3-A4, CYP3-B4,* and *VEGFA* gene expression in HepaRG cells and *CYP1-A1* and *VEGFA* in HepG2 cells significantly. For instance, 95% of absorbed xylene is metabolized in the liver [27]. However, hepatotoxicity is varied across other C9 alkylbenzenes, where ethylbenzene shows hepatotoxicity caused by oxidative stress [28], while xylene, trimethyl benzenes, and toluene show mild, potentially adaptive effects (www.atsdr.cdc.gov accessed: 22 August 2024) (https://www.ncbi.nlm.nih.gov/books/NBK241481/ accessed: 20 September 2024) [29], suggesting that C9 alkylbenzenes potentially target liver and liver may regulate fatty liver diseases. Our data demonstrated that exposure to 2-ET or in a combination of 3-ET and 4-ET increased *FGF-23*, *Cyt-7,* and *TGF-β* in HepaRG cells, and exposure to 2-ET, 3-ET, and 4-ET increased p21, MMP2, and TGF-β in HCC HepG2 cells. Increased expression of p21 (a cell cycle inhibitor) is associated with liver fibrosis that can contribute to the progression of liver diseases such as nonalcoholic fatty liver disease (NAFLD) and alcoholic liver disease (ALD) [30]. Therefore, our data suggest that ETs regulated cell fibrosis signaling in liver cells.

## 5. Conclusions

In conclusion, for the first time, we showed that ETs induced cell toxicity, ROS production, inflammatory gene expression, altered mitochondrial respiration, and upregulates drug metabolic *CYP1-A1, CYP1-A2 CYP2-D6, CYP2-E1, CYP3-A4, CYP3-B4,* and *VEGFA* gene expression in HepaRG and HCC HepG2 and SK-Hep1 cells. Acute exposure of 2-ET, 3-ET, and 4-ET, one or in combination, and chronic 2-ET exposure upregulated cell fibrosis markers such as *AST, FGF-23, Cyt-7 p21, TGFβ, TIMP2,* and *MMP2* in liver cell models, suggesting that ETs target liver cells (Figure 6C) and may dysregulate liver function and increase risk of liver diseases such as NAFLD.

## Data Availability

The published article includes all data sets generated/analyzed for this study. Data will be made available upon request.

## Supplementary Materials

The following supporting information can be downloaded at https://www.mdpi.com/article/10.3390/toxics12120856/s1, Figure S1: ETs induced ROS production in liver cell model. Figure S2: ETs exposure affects mitochondrial OXPHOS, glycolysis, and cell energy phenotype in liver cells. Figure S3: ET exposure modulates cell steatosis in liver cells. Table S1: Set of primers used in the current study.
