# Supplementary material for: Ethyltoluenes Regulate Inflammatory and Cell Fibrosis Signaling in the Liver Cell Model
Source: Toxics. 2024 Nov 27;12(12):856. doi: 10.3390/toxics12120856 (PMC11679060; doi:10.3390/toxics12120856)
Supplement: Supplementary file 1 [file toxics-12-00856-s001.zip › toxics-3295326-supplementary.pdf]

Supplement Fig. S1

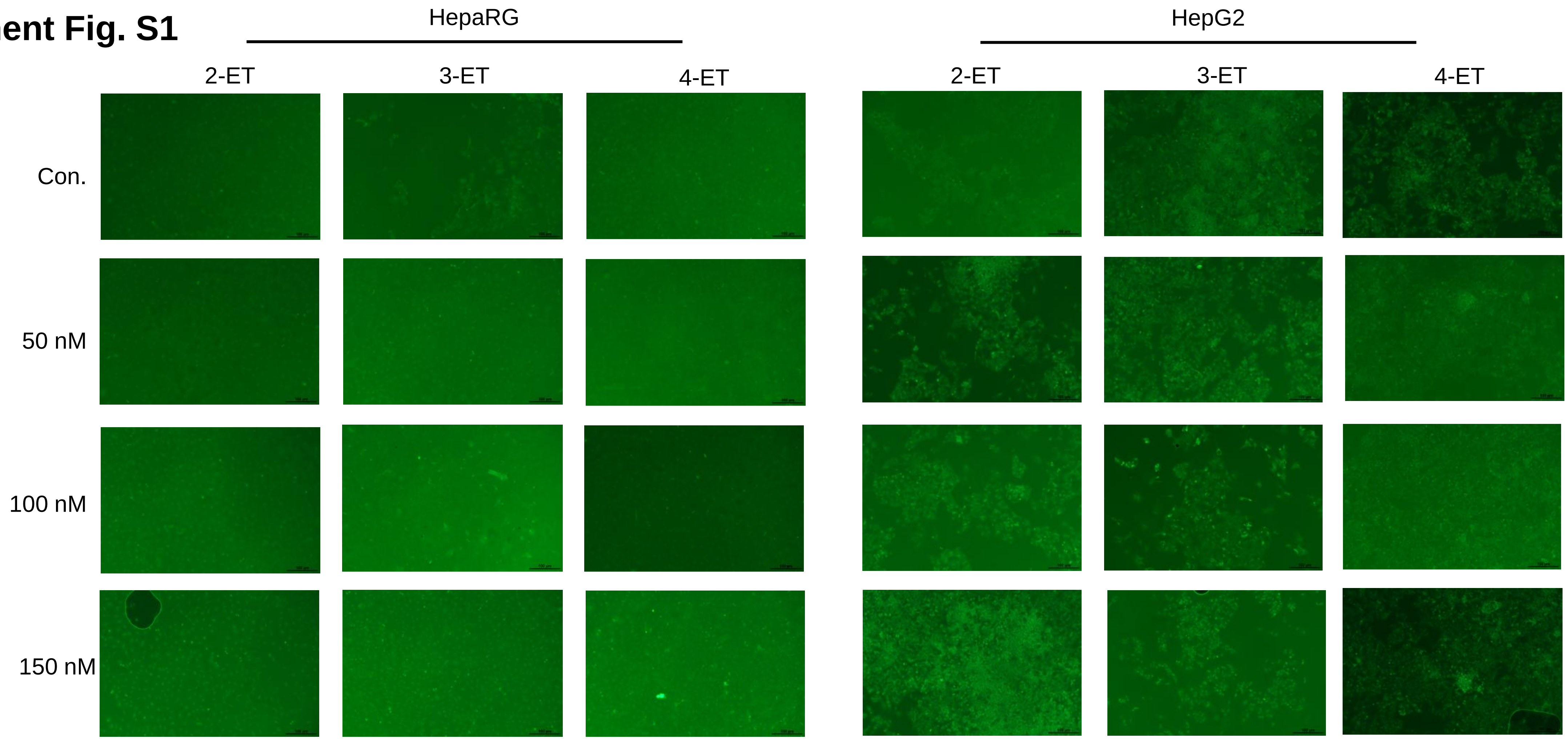

Figure S1: ETs induced ROS production in Liver cell model. HepaRG and HepG2 cells were cultured in 96 well plates in triplicates for 12 h and cells were treated with ETs (50-250 nM) for 72 h. Cells were further treated with CellRox green reagent and then observed under Keyence BZX-810 fluorescence microscope (10X objective) and images were captured.

Supplement Fig. S2

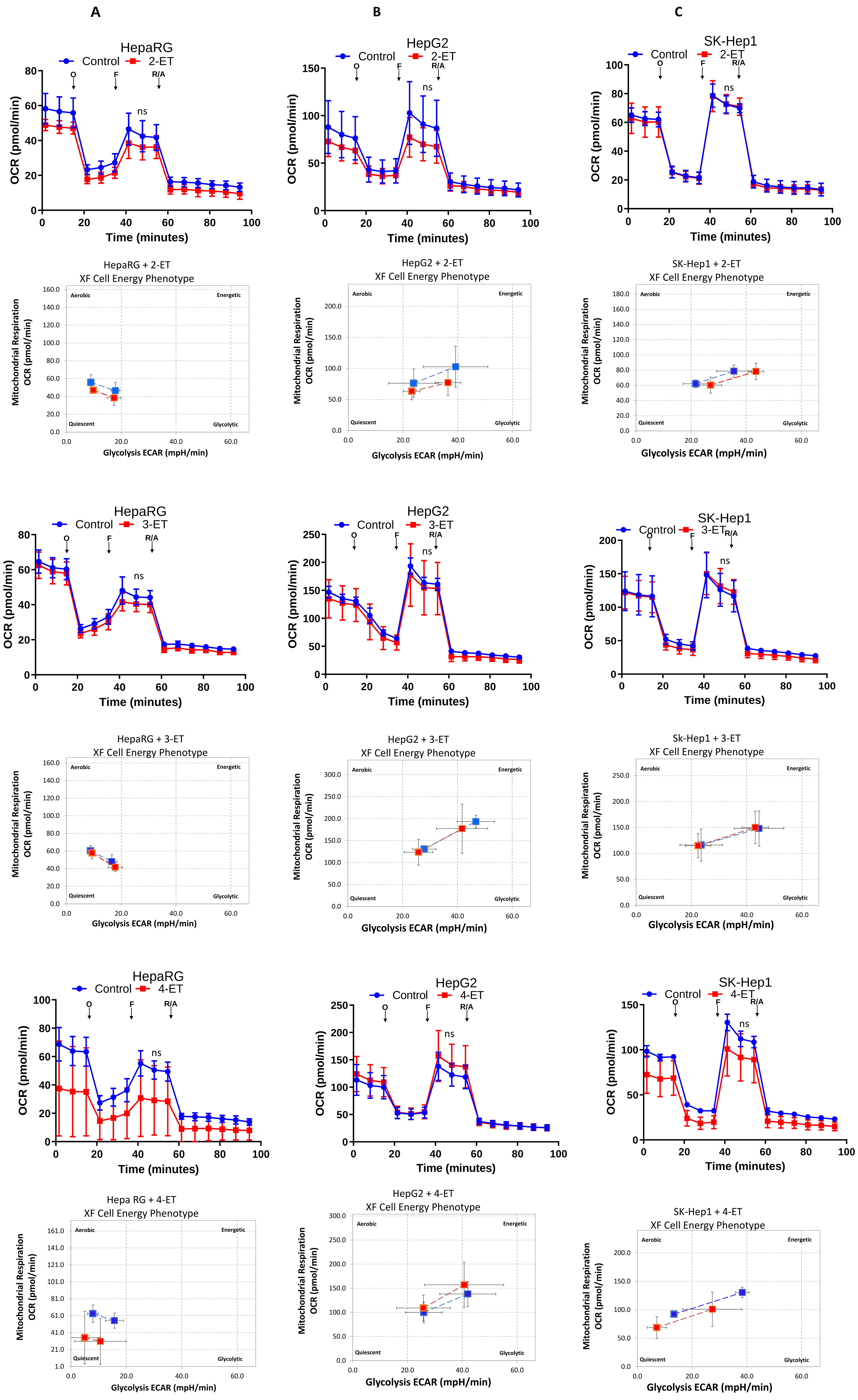

Figure S2: ET exposure affects mitochondrial OXPHOS, glycolysis, and cell energy phenotype in liver cells. A-C) HepaRG and HCC HepG2 and SK-Hep1 cells were subjected to Seahorse Bio-analyzer, and relative OXPHOS and glycolysis were analyzed as described in the materials and methods section (Left, right and middle panels). ns- not significant compared to control cells. O- Oligomycin. F- FCCP. R/A- Rotenone and Antimycin.

Supplement Fig. S3

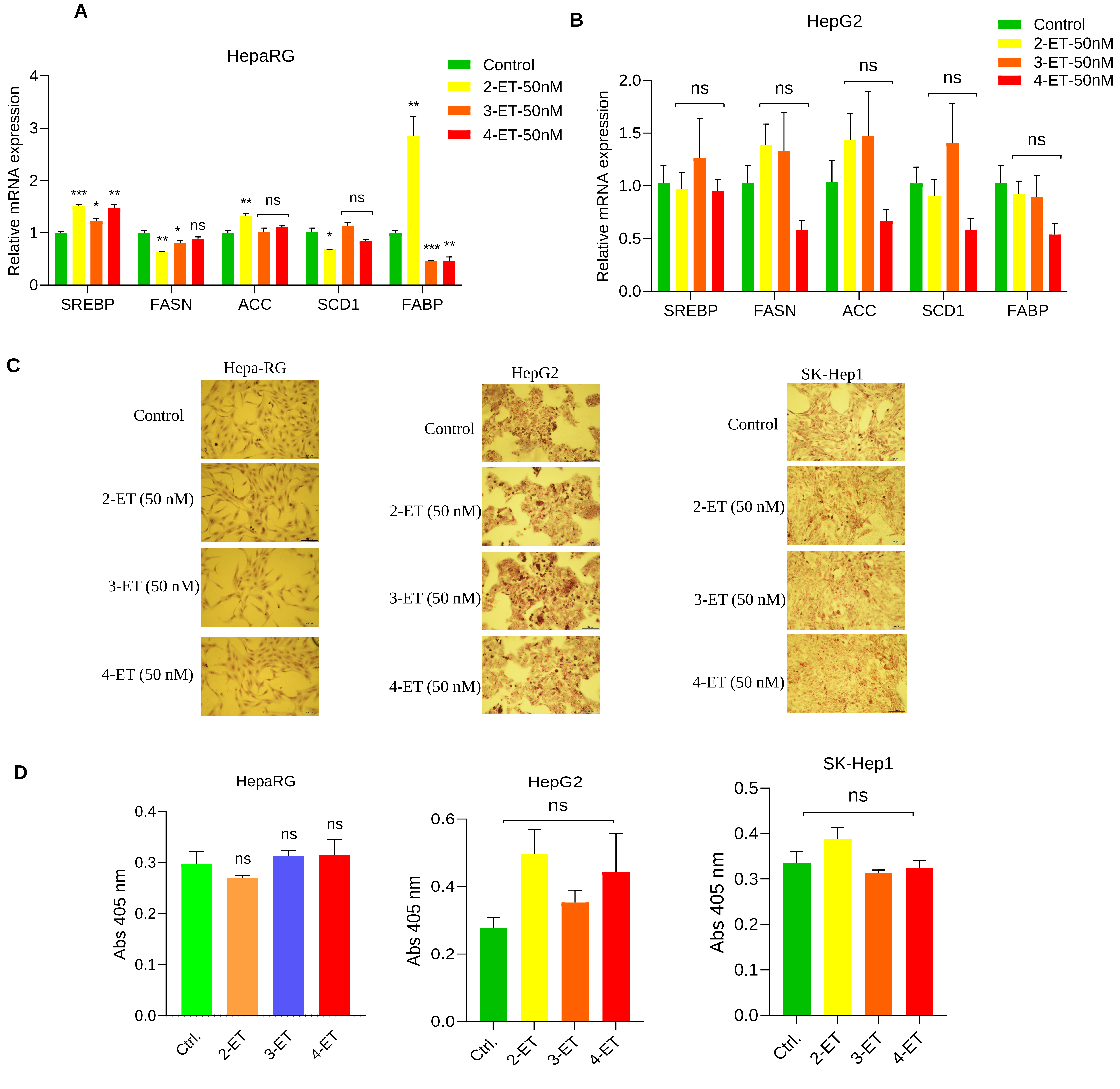

Figure S3: ET exposure modulates cell steatosis in liver cells. A&B) HepaRG and HepG2 cells were treated with ETs (50nM) for 72 h. The expression of lipogenic genes were analyzed by RT/qPCR as described in the materials and methods section. *$P < 0.05$, **$P < 0.01$, and ***$P < 0.001$ compared with untreated cells. ns -not significant. **C)** HepaRG, HepG2, and SK-Hep1 cells were grown on coverslips and treated with ETs (50nM) for 72h and then cells were treated with 100 μM oleic acid for 24h in presence of ETs. Cells were fixed, stained with Oil Red O (ORO), and cell images were captured using a Nikon Y-IDP microscope and presented. **D**) HepaRG, HepG2 and SK-Hep1 cells (1×10^4/well) were grown in 96-well plates in triplicates, treated with ETs for 30h, and then treated with 100 μM oleic acid (Sigma) for 24h. Cells were fixed with paraformaldehyde (4%) and stained with Oil Red O. Cells were lysed in 100 μl of 1X cell lysis buffer and Oil Red O stain released from steatotic cells was transferred to another 96-well plate. The absorbance at 405 nm was measured using a Fluostar Omega plate reader (BMG Lab Tech, Cary, NC) as described in the materials and methods section.

Supplementary Table 1

Set of primers used in the current study.

**Primer sequence**
GAPDH FP: 5’-CCACCCAGAAGACTGTGGAT -3’<br>GAPDH RP: 5’-GTTGAAGTCAGAGGAGACCACC-3’
SREBP1 FP: 5’-GAGCCATGGATTGCACTTTC-3’<br>SREBP1 RP: 5’-AGCATAGGGTGGGTCAAATAG-3’
L-FABP FP: 5’-GGAATGTGAGCTGGAGACAA-3’<br>L-FABP RP: 5’-AGTTCGGTCACAGACTTGATG-3’
SCD1 FP: 5’-CTCTTTCTGCTCTGCCATCTT-3’<br>SCD1 RP: 5’-CCCGACTTCACCTCCTTAAATC-3’
FASN FP: 5’-CTAGGTTTGATGCCTCCTTCTT-3’<br>FASN RP: 5’-GATGGCTTCATAGGTGACTTCC-3’
ACC FP: 5’-GCAGGTCACACGTCTCTTTAT-3’<br>ACC RP: 5’-CCAGCCTGTCATCCTCAATATC-3’
TIPM2 FP: 5’-CCGAGACAAAGAGGAGAGAAAG -3’<br>TIMP2 RP: 5’-CCTCCTGATACGGGTGCCATAA -3’
MMP2 FP: 5’-CCGTGGTGAGATCTTCTTCTTC-3’<br>MMP2 RP: 5’-GGTTCTCCAGCTTCAGGTAATAG-3’
AST FP: 5’-CGAATTTTGAGACTGGGATTAGA -3’<br>AST RP: 5’-CACTCTGGGTTGAGATGATGAA -3’
TGFβ FP: 5’- AGGTTATTTCCGTGGGATACTG-3’<br>TGFβ RP: 5’-CGATAGTCTTGCAGGTGGATAG -3’
p21 FP: 5’-CGCTCTACATCTTCTGCCTTAG -3’<br>p21 RP: 5’- CGGGATGAGGAGGCTTTAAATA-3’
Cytokeratin7 FP: 5’-GCTGTCCGGTGGTTAATTTC-3’<br>Cytokeratin7 RP: 5’-CAACAAGTTTTCCTCCCTCATC -3’
FGF23 FP: 5’-TAACCCATCCCTCAGCAAAC -3’<br>FGF23 RP: 5’-GAGAGGCACAAGGAAGAGAAATA -3’
CAT FP: CCTCTCATCCCAGTTGGTAAAC<br>CAT RP: TGTTGAATCTCCGCACTTCTC
SOD1FP: GTGTGGCCGATGTGTCTATT<br>SOD1RP CTCAGACTACATCCAAGGGAATG
CYP1A1 FP: GGTCTTTCTCTTCCTGGCTATC<br>CYP1A1 RP: CTGTCTCTTCCCTTCACTCTTG
CYP1A2 FP: CAGGAGCACTATCAGGACTTTG<br>CYP1A2 RP: GTTGACCTGCCACTGGTTTA
CYP3B4 FP: CTGAGAAGTTCCTCCCTGAAAG<br>CYP3B4 RP: AGAAGCAGAGGAGCCAAATC
CYP2E1 FP: CTGAGAAGTTCCTCCCTGAAAG<br>CYP2E1 RP: AGAAGCAGAGGAGCCAAATC
CYP3A4 FP: CTGAGAAGTTCCTCCCTGAAAG<br>CYP3A4 RP: AGAAGCAGAGGAGCCAAATC
CYP2D6 FP: CTAAGGGAACGACACTCATCAC<br>CYP2D6 RP: AGGGAGGTGAAGAAGAGGAA

NAT1(3) FP: GATGTGAACTGCAACTCCAAAG<br>NAT1(3) RP: GCCCAGTACAGAAGATGATTGA
IL1B FP: CATGGGATAACGAGGCTTATGT<br>IL1B RP: CCCAAGGCCACAGGTATTT
STAT3 FP: CTTGGGACCTGGTGTGAATTA<br>STAT3 RP: CCTTGGGAATGTCAGGATAGAG
HMOX1FP: GGTCCTTACACTCAGCTTTCT<br>HMOX1RP: CATAGGCTCCTTCCTCCTTTC
CXCL8(IL8) FP: AGACAGCAGAGCACACAAG<br>CXCL8(IL8) RP: GGGTGGAAAGGTTTGGAGTAT
VEGFA FP: TGGTGTCTTCACTGGATGTATTT<br>VEGFA RP: CCTGTACCTGTGATCTGTCTTTC
IL6 FP: 5’-GTAGTGAGGAACAAGCCAGAG-3’<br>IL6 RP: 5’-GGACTGCAGGAACTCCTTAAA-3’
TNFα FP: 5’- GAGCCAGCTCCCTCTATTTATG-3’<br>TNFα RP: 5’- AGGGCGATTACAGACACAAC-3’
